# Comprehensive Review and Assessment of Computational Methods for Prediction of N6-Methyladenosine Sites

**DOI:** 10.3390/biology13100777

**Published:** 2024-09-28

**Authors:** Zhengtao Luo, Liyi Yu, Zhaochun Xu, Kening Liu, Lichuan Gu

**Affiliations:** 1 School of Information and Artificial Intelligence, Anhui Agricultural University, Hefei 230036, China; lzt9781@163.com; 2 Anhui Provincial Key Laboratory of Smart Agriculture Technology and Equipment, Anhui Agricultural University, Hefei 230036, China; 3 Computer Department, Jingdezhen Ceramic University, Jingdezhen 333403, China; yuliyi@jcu.edu.cn (L.Y.); zhaochunxu@hrbmu.edu.cn (Z.X.); 4 School for Interdisciplinary Medicine and Engineering, Harbin Medical University, Harbin 150076, China

**Keywords:** m^6^A site identification, deep learning, machine learning, performance evaluation, performance evaluation, independent test datasets

## Abstract

**Simple Summary:**

This study provides a comprehensive review and evaluation of computational methods for the prediction of N6-methyladenosine (m^6^A) sites, crucial in regulating cellular functions and gene expression. Advances in high-confidence m^6^A site mapping have enabled the development of robust computational approaches. We assess 52 computational methods, including machine learning, deep learning, and ensemble-based techniques, using 13 benchmark datasets from nine different species. The evaluation reveals that deep learning methods generally surpass traditional scoring function-based approaches. This systematic analysis aims to guide the design and refinement of computational tools for m^6^A identification, facilitating rigorous method comparison and supporting future research in RNA modifications. The findings are vital in understanding m^6^A-dependent mRNA regulation, with implications in addressing diseases like cancer, where m^6^A plays a regulatory role.

**Abstract:**

N6-methyladenosine (m^6^A) plays a crucial regulatory role in the control of cellular functions and gene expression. Recent advances in sequencing techniques for transcriptome-wide m^6^A mapping have accelerated the accumulation of m^6^A site information at a single-nucleotide level, providing more high-confidence training data to develop computational approaches for m^6^A site prediction. However, it is still a major challenge to precisely predict m^6^A sites using in silico approaches. To advance the computational support for m^6^A site identification, here, we curated 13 up-to-date benchmark datasets from nine different species (i.e., *H. sapiens*, *M. musculus*, *Rat*, *S. cerevisiae*, *Zebrafish*, *A. thaliana*, *Pig*, *Rhesus*, and *Chimpanzee*). This will assist the research community in conducting an unbiased evaluation of alternative approaches and support future research on m^6^A modification. We revisited 52 computational approaches published since 2015 for m^6^A site identification, including 30 traditional machine learning-based, 14 deep learning-based, and 8 ensemble learning-based methods. We comprehensively reviewed these computational approaches in terms of their training datasets, calculated features, computational methodologies, performance evaluation strategy, and webserver/software usability. Using these benchmark datasets, we benchmarked nine predictors with available online websites or stand-alone software and assessed their prediction performance. We found that deep learning and traditional machine learning approaches generally outperformed scoring function-based approaches. In summary, the curated benchmark dataset repository and the systematic assessment in this study serve to inform the design and implementation of state-of-the-art computational approaches for m^6^A identification and facilitate more rigorous comparisons of new methods in the future.

## 1. Introduction

N6-methyladenosine (m^6^A), the best-characterized RNA modification, is formed by the methylation of adenosine at position 6. This modification is the most abundant within mRNA in mammalian cells [1] and could be also found in primary miRNA (pri-miRNA) [2], long intergenic ncRNAs (lincRNAs) [3], and rRNA [4]. m^6^A has recently been shown to alter the nuclear export of RNA, RNA splicing, RNA stability, and translation efficiency [5]; moreover, it has been demonstrated to link to a wide range of cellular functions [6,7,8,9,10,11,12,13]. The aberrant regulation of m^6^A has also been implicated in a variety of cancers, such as lung cancer, liver cancer, breast cancer, glioblastoma, and acute myeloid leukemia [14,15], highlighting the crucial regulatory roles of m^6^A modification. Consequently, the precise identification of m^6^A modification sites at the transcriptome level is of vital importance to understand and explore underlying m^6^A-dependent mRNA regulation mechanisms and biological functions.

A variety of sequencing techniques have been developed for transcriptome-wide m^6^A mapping, spanning three important periods from non-single-nucleotide resolution to single-base resolution in bulk cellular populations to single-base resolution within single cells. During its first developmental period, methylated RNA immunoprecipitation and sequencing such as MeRIP-seq or m^6^A-seq [16] emerged as a classical technique widely used to detect m^6^A. However, this mapping technique only localizes m^6^A residues to approximate regions with a ~100 nucleotide (nt) length, and it cannot identify the exact positions of individual m^6^A sites at a transcriptome-wide level. This disadvantage was addressed during the second development period, when a variety of refined methods were developed to improve the resolution for whole-transcriptome m^6^A identification and quantification, such as PA-m^6^A-seq [17], miCLIP [18], UV-CLIP [19], m^6^A-REF-seq [20], and DART-seq [21]. Recently, a novel method called scDART-seq [22] has been developed to identify single-nucleotide m^6^A sites at a transcriptome-wide level in single cells, marking the advent of a more sophisticated m^6^A profiling era. Although such advanced experimental methods are too time-consuming and costly to perform genome-wide analysis, they open the door to research on precise m^6^A mapping and provide sufficient data for the development of in silico methods of m^6^A site identification.

During the past few years, a number of computational approaches have been developed for the prediction of m^6^A sites in the RNA of different species, such as *H. sapiens*, *M. musculus*, *Chimpanzee*, *Rhesus*, *Pig*, *Rat*, *Zebrafish*, *D. melanogaster*, *S. cerevisiae*, and *A. thaliana* (Supplementary Material Figure S1A). Such computational methods can be generally categorized into three groups according to their computational methodologies, including (i) traditional machine learning-based methods (accounting for 60%), (ii) deep learning-based methods (24%), and (iii) ensemble learning-based methods (16%) (Supplementary Material Figure S1B). Several surveys [23,24,25] of RNA methylation prediction have been published (Supplementary Material Figure S1C); however, there is almost no comprehensive and specific overview of m^6^A site prediction. Only one focuses on tools published prior to 2019 for the prediction of m^6^A sites in the RNA of *S. cerevisiae* [24]. More recent tools for *S. cerevisiae*, as well as predictors of other species, have not been systematically reviewed; in particular, there is a lack of comprehensive assessments of the prediction performance of the compared approaches via the implementation of extensive and independent benchmarking tests.

To overcome the issues mentioned above, here, we conduct a comparative and systematic analysis by summarizing the most up-to-date research progress in m^6^A site prediction. For this purpose, we have manually curated 13 up-to-date and large-scale benchmark datasets with high-confidence single-nucleotide m^6^A site sequences to accompany this comprehensive survey analysis, thereby helping the community to carry out an unbiased evaluation of alternative approaches and support future research on m^6^A modification. A total number of 52 computational methods, collected from Web of Science and PubMed with the keywords ‘m^6^A prediction’ or ‘m^6^A identification’, are carefully assessed, benchmarked, and extensively discussed in terms of feature extraction, model construction, performance evaluation strategies, and webserver/software usability. More importantly, using the 13 independent test datasets, which have never been seen in the training data of the existing approaches, we have systemically evaluated the generalization and robustness of the investigated methods with available online webservers or locally stand-alone software. We expect that the comparative analysis in this study will serve as a critical analysis of state-of-the-art m^6^A prediction approaches and pave the way for future development to accurately predict m^6^A sites in single cells.

The structure of this paper consists of four main parts. The first part provides an overview of the research background and significance of m^6^A site prediction, introducing existing sequencing technologies and computational methods. The second part systematically compares computational methods for m^6^A site prediction, including the construction of training datasets, feature engineering, and prediction algorithms. The third part presents the experimental results, covering species-specific predictions, cross-species validation, and the performance comparison of single-cell m^6^A site prediction. The fourth part concludes with a summary of the research findings and considerations for future work.

## 2. Systematic Comparison of Computational Approaches for m^6^A Site Prediction

### 2.1. Existing Methods for m^6^A Site Prediction

Among the 52 computational approaches for m^6^A site prediction analyzed in this study, 32 were designed and implemented based on m^6^A data at a non-single-nucleotide resolution. For the prediction of m^6^A sites in the *S. cerevisiae* genome, Chen et al. constructed a balanced dataset termed Smet1307, which consisted of 1307 positive samples and 1307 negative samples with a 51 nt length after removing sequence redundancy. They proposed the first computational predictor, iRNA-Methyl [26], in this field. To further improve the prediction accuracy, scholars have developed several predictors based on machine learning, such as support vector machine (SVM) [27,28,29,30,31,32], random forest (RF) [33], and eXtreme Gradient Boosting (XGBoost) [34], as well as ensemble learning [35,36], using various sequence-based feature extraction methods. Additionally, two SVM-based predictors [37,38] were successively developed based on a high-confidence subset, named Smet1307sub, which was generated after filtering m^6^A sites with distances to the detected m^6^A-seq peaks greater than 10 bp from the dataset Smet1307. Recently, a new deep learning-based predictor named iMethyl-Deep [39] was proposed using Smet1307 and another dataset, Smet3270 [24], which contained 3270 experimentally verified m^6^A sites and 3270 non-m^6^A sites.

For the prediction of m^6^A sites in the RNA of H. sapiens, Chen et al. first developed an SVM-based model called iRNA-PseColl [40] using a dataset, Hmet1130, which consisted of 1130 experimentally verified positive samples and 1130 negative samples with a 41 nt length. Moreover, this predictor could implement other prediction functions for m1A and m5C sites after being trained on the corresponding benchmark dataset. Based on Chen’s data, a deep learning-based model called iRNA-Mod-CNN [41] was proposed to further improve the prediction performance for m^6^A sites in the RNA of *H. sapiens*. For the identification of m^6^A sites in the *A. thaliana* transcriptome, Chen et al. developed an SVM-based predictor, M6ATH [42], based on a dataset, Amet394, that contained 394 experimentally verified m^6^A sites and 394 non-m^6^A sites. In the same year, a new model called AthMethPre [43] was developed on another larger dataset that covered 5081 experimentally verified positive samples and 5081 negative samples. Additionally, another 14 computational approaches [44,45,46,47,48,49,50,51,52,53,54,55,56,57] were proposed using m^6^A data at a non-single-nucleotide resolution from multiple species.

Seventeen computational approaches for m^6^A site prediction analyzed in this study were proposed based on single-nucleotide m^6^A data. Such computational models can be categorized into two types, namely species-specific predictors [58,59,60,61,62,63,64,65,66,67,68,69] and tissue-specific predictors [70,71,72,73,74]. Four public databases, namely RMVar [75], RMBase [76,77], Met-DB [78,79], and the Ensembl database “http://www.ensembl.org (accessed on 12 September 2022)”, were used as the mainstream data resources for training dataset construction for species-specific predictors, while the latest data generated by m^6^A-REF-seq [20] were used for the training of tissue-specific predictors. Significantly, among these predictors, MASS [68] and MultiRM [58] are the two most important and recommended methods in view of their innovation. MASS, a multi-task framework embedding a convolutional neural network (CNN) and bi-directional long short-term memory (BiLSTM), is the first computational method based on a multi-task curriculum learning strategy for the extraction of shared sequence features across multiple species in the prediction of m^6^A sites simultaneously. MultiRM, an attention-based multi-label deep learning framework, is the first computational model aimed at simultaneously predicting the putative sites of twelve widely occurring transcriptome modifications, including m^6^A modifications.

**Figure 1:**
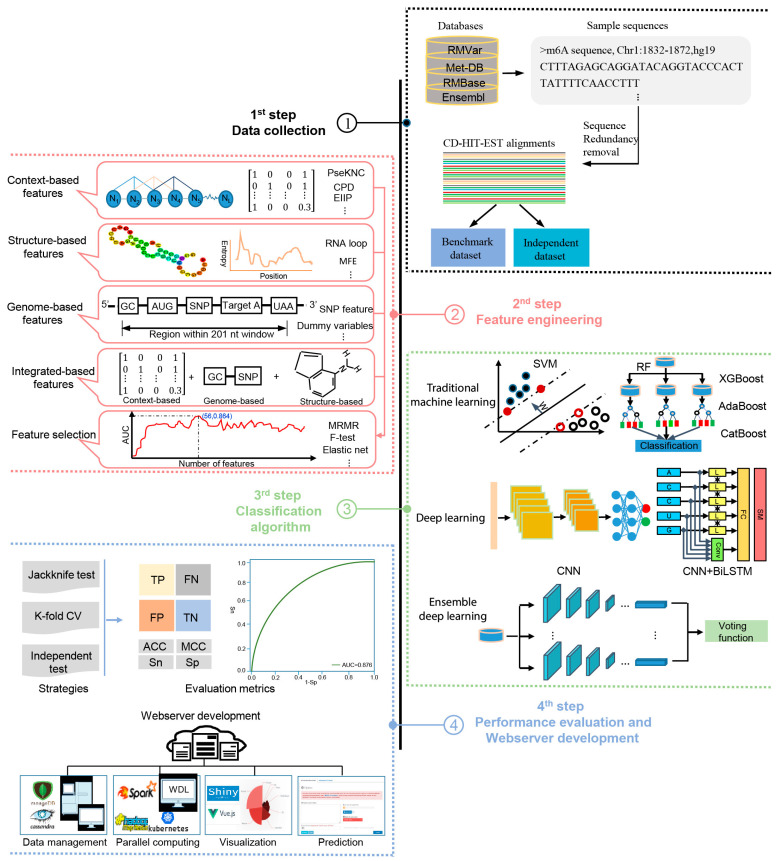
A graphical illustration of four common steps in the construction and evaluation of computational approaches for the prediction of m^6^A sites.

The methods analyzed in this study are systematically described and summarized in Table 1 in terms of the targeted species, sequence length, algorithm, features, evaluation strategy, webserver availability, and data size. The four generic steps used by these computational approaches to identify m^6^A sites are illustrated in Figure 1; these will be discussed in detail in the following sections.

### 2.2. Construction of Training Dataset

As mentioned above, the data used for training and benchmarking in m^6^A site prediction are composed of non-single-nucleotide data collected from the literature and single-nucleotide data derived from public databases (Table 2). The generic steps to construct the training dataset for m^6^A site prediction are given as follows. (i) m^6^A site information including the chromosome type, m^6^A site location, strand sequence, species, and reference genome version was collected from the literature or public databases. (ii) Based on such m^6^A site information, a (2ξ+1)-nt-long sliding window was used to extract sample sequences with adenosine at the center along each of the RNA segments. Only m^6^A sites containing the motif DRACH (where D = A, G or U, R = A or G, H =A, C, or U) were retained as positive samples, while those unmethylated adenosines with the motif DRACH along the whole transcriptome were used as negative samples. (iii) Subsequently, the CD-HIT-EST tool [83] was employed to remove those sequences with sequence similarity greater than a certain threshold (typically 80%) to reduce the redundancy in the sample sequences. (iv) Finally, negative samples were randomly sampled as a certain positive-to-negative ratio to construct a balanced or unbalanced dataset. (v) Generally, the obtained dataset could be divided into two subsets, including a training dataset and testing dataset. This construction process for training datasets could be also applicable to other post-transcriptional modifications and protein post-translational modifications.

**Table 2:** Detailed information of public databases.

Database	Species	Latest Version	Feature	Website (URL)
Met-DB [78,79]	*H. sapiens*, *M. musculus*	1.0 (November 2014)	MeT-DB is the first comprehensive resource for m^6^A in the mammalian transcriptome and provides ∼300 k m^6^A methylation sites in 74 MeRIP-Seq samples from 22 different experimental conditions.	http://compgenomics.utsa.edu/methylation/ (accessed on 12 September 2022)
RMBase [76,77]	*H. sapiens*, *M. musculus*, *Rhesus*, *Chimpanzee*, *Rat*, *Pig*, *Zebrafish*, *S. cerevisiae*, *Fly*, *A. thaliana*, *S. pombe*, *E. coli*, *P. aetuginosa*	2.0 (October 2017)	RMBase v2.0 was expanded with ∼600 datasets and ∼1,397,000 modification sites from 47 studies among 13 species, including ∼1,373,000 m^6^A sites at a single nucleotide or very high resolution.	http://rna.sysu.edu.cn/rmbase/ (accessed on 12 September 2022)
RMVar [75]	*H. sapiens*, *M. musculus*	2.0 (October 2020)	RMVar is an updated version of m6Avar and contains 179,270 high-confidence m^6^A sites from *H. sapiens* and 10,760 from *M. musculus* in total.	http://rmvar.renlab.org (accessed on 12 September 2022)
m6A-Atlas [84]	*H. sapiens*, *M. musculus*, *A. thaliana*, *Fly*, *Rat*, *Yeast*, *Zebrafish*, *virus*	1.0 (August 2020)	m6A-Atlas is a comprehensive knowledge base for the unraveling of the m^6^A epitranscriptome and provides 442,162 high-confidence m^6^A sites identified from seven base-resolution technologies.	www.xjtlu.edu.cn/biologicalsciences/atlas (accessed on 12 September 2022)
ConsRM [65]	*H. sapiens*	1.0 (February 2021)	ConsRM is a database on the collection and large-scale prediction of evolutionarily conserved RNA methylation sites and includes 177,998 base-resolution human m^6^A RNA methylation sites with ConsRM scores.	https://www.xjtlu.edu.cn/biologicalsciences/con (accessed on 12 September 2022)
Ensembl	*H. sapiens*, *M. musculus*	106 (April 2022)	Ensembl annotates genes, collects disease data, and provides m^6^A site information from mammalian species.	https://asia.ensembl.org/index.html (accessed on 12 September 2022)

### 2.3. Construction of Independent Test Dataset

In order to objectively evaluate the generalization performance of the current and existing methods, we collected single-base-resolution m^6^A sites recently validated by experiments and then constructed a total of 13 balanced independent test datasets after removing the overlapping sequences with the training datasets of the compared methods. The resulting independent test datasets contained sequences for eukaryotes, including *H. sapiens*, *M. musculus*, *Rat*, *S. cerevisiae*, *Zebrafish*, *A. thaliana*, *Pig*, *Rhesus*, and *Chimpanzee*. Herein, we describe the detailed procedures for the curation of the independent test datasets (Table 3).

Specifically, we collected m^6^A site sequences containing the motif DRACH with a consistent length with the above-mentioned computational approaches from the recent literature and according to m^6^A site information produced by m^6^A-seq2 [85], m^6^A-RFE-seq [86], m^6^A-SAC-seq [87], miCLIP [88], and scDART-seq [22], respectively. Moreover, the m^6^A site sequences with a single-base resolution deposited in the recently published database m6A-Atlas [84] were also included. After removing those sequences with sequence similarity greater than 80% using CD-HIT-EST and further removing the overlapping sequences with positive samples of the training datasets of the compared methods, the remaining sequences were retained as the positive samples of the independent test datasets.

Non-m^6^A sequences (i.e., negative samples) refer to RNA sequences of which the central positions are confirmed not to be m^6^A sites. In accordance with the approaches for the selection of negative samples in the majority of studies about m^6^A site prediction, all possible non-m^6^A sequences containing unmethylated adenosines in the motif DRACH at sequence centers could be extracted from the reference genomes of species corresponding to positive samples. Next, the overlapping sequences with the training datasets of the compared methods, as well as the positive samples of the independent test datasets, were removed, thereby enabling the remaining sequences to not be seen in these two types of datasets. As the distribution of m^6^A sites and non-m^6^A sites in the transcriptome is unbalanced, after removing those sequences with greater than 80% similarity, the non-m^6^A sequences were randomly selected from the remaining sequences in terms of a certain positive-to-negative ratio to construct balanced and unbalanced independent test datasets. In the current study, the positive-to-negative ratio was set to 1:1. The numbers of non-m^6^A sequences in the independent test datasets for seven species are shown in Table 3.

**Table 3:** Detailed information of independent test datasets.

Species	Dataset Name	Source	Positive-to-Negative Ratio
			1:1
*H. sapiens*	*Hg38_Human*	scDART-seq data containing single-nucleotide m^6^A sites in single cells [22]	22,248
	*hg19_Human1*	Single-nucleotide m^6^A data [86]	2064
	*hg19_Human2*	Single-nucleotide m^6^A data [85]	37,372
	*hg19_Human3*	Single-nucleotide m^6^A data [88]	930
	*hg19_Human4*	Data intersection between ConsRM, RMBase, and m6A-Atlas	12,588
*M. musculus*	*mm10_Mouse*	Data intersection between RMVar, RMBase, and m6A-Atlas	3330
*Rhesus*	*rheMac8_Rhesus*	RMBase	12,098
*Chimpanzee*	*panTro4*_*Chimpanzee*	RMBase	15,424
*Rat*	*rn5_Rat*	RMBase	24,380
*Pig*	*susScr3_Pig*	RMBase	42,838
*Zebrafish*	*danRer10_Zebrafish*	RMBase	8946
*S. cerevisiae*	*sacCer3_S_cerevisiae*	Data intersection between m6A-Atlas and RMBase	14,876
*A. thaliana*	*TAIR10_A_thaliana*	Data intersection between m6A-Atlas and RMBase	4516

### 2.4. Feature Engineering and Representation

To develop robust and reliable computational approaches for m^6^A prediction, it is vitally important to carefully design and extract features for the representation and conversion of the RNA sequences into numeric vectors. The features applied in the 52 computational approaches that we revisited in this study can be categorized into four groups, i.e., context-, structure-, genome-based, and integrated features.

#### 2.4.1. Context-Based Features

Context-based features are designed and extracted to describe the genomic contexts of the m^6^A/non-m^6^A sequences. These context-based features of the 52 approaches systematically described and summarized in Table 1 can be classified into three groups: (i) nucleotide physicochemical properties, such as the pseudo-dinucleotide composition (PseDNC) [26,30,35,55], general PseDNC [44], pseudo-trinucleotide composition (PseTNC) [45], dinucleotide-based auto-/cross-covariance (DAC/DCC) [27,29,32,70], chemical property with density (CPD) [33,37,40,42,46,47,54,59,70], and electron–ion interaction pseudopotential (EIIP) [53,72]; (ii) RNA primary sequence-derived features, including one-hot or binary encoding [31,39,54,56,58,66,67,68,71,74], RNA word embedding [49,50,67,69], dinucleotide binary encoding [57], the k-mer composition [33,34,41,43,45,52,62], the KNN score [28,45], features related to entropy information [60], and the local position-specific dinucleotide frequency (LPSDF) [57]; and (iii) position-specific scoring matrices, such as the nucleotide pair position specificity (NPPS) [48,53], position-specific k-mer nucleotide propensity (PS(k-mer)NP) [34,36,38,64,73], probability matrix [51], and bi-profile Bayes (BPB) [53].

Context-based features are the most widely applied feature type for m^6^A site prediction. Most of these features can be easily generated by useful bioinformatics pipelines without following complex mathematical formulas, such as Pse-in-One [89], iFeature, PseKNC [90], PseKNC-General [91], iLearn [92], and iLearnPlus [93]. Among the above-mentioned features, the chemical property with density (CPD), which describes three distinct structural chemical properties of nucleotides, including functional groups, ring structures, and hydrogen bonds, and the density of the *i*-th nucleotide along the sample sequence, have been extensively used in computational biology [94,95,96,97]. Compared to earlier published models, the majority of the computational methods for the identification of m^6^A sites since 2019 have favored binary encoding features.

#### 2.4.2. Structure-Based Features

m^6^A/non-m^6^A sequences can also be characterized by structure-based features, which are calculated based on 3D RNA structures. Several structure-based features have been applied to four predictors, namely WHISTLE [59], RNAMethPre [62], SRAMP [63], and ConsRM [65], and have been confirmed to be effective in improving their prediction performance. These structure-based features, predicted by the RNAfold [98] tool, can be categorized into two major types: (i) the predicted RNA loop region [59,63,65], such as bulged loop (B), hairpin loop (H), interior loop (I), multiple loop (M), and paired (P), which can be encoded into the binary vectors (0,0,0,0,1), (1,0,0,0,0), (0,0,1,0,0), (0,1,0,0,0), (0,0,0,1,0), respectively; (ii) the minimum free energy (MFE) [62], which is calculated to measure the secondary structure strength of the region around the site; and (iii) the predicted RNA hybridized region [59,65], which is computed by minimizing the free energy.

#### 2.4.3. Genome-Based Features

Genome-based features [59,60,62,65] can contribute to m^6^A site prediction because such features may capture the adequate attributes of the m^6^A modification topology. In total, there are eight major types of genome-based features, namely (i) dummy variables indicating whether the site is overlapped with the topological region on the major RNA transcript, such as the 5′ UTR, 3′ UTR, coding sequence, stop/start codons flanked by 100 bp, downstream 100 bp of TSS on A, and so on; (ii) the relative position in the region, including the relative position in the 5′ UTR/3′ UTR, the relative position in the coding sequence, and the relative position in an exon; (iii) the nucleotide distances to the splicing junctions, such as the distance to the 5′/3′ splicing junction; (iv) the region length, including the 5′ UTR/3′ UTR length, coding sequence length, gene length in exons, full gene length, mature transcript length, and full transcript length; (v) clustering information, such as the count of neighboring input sites at 1001/101 nt, the count of neighboring As within a 2001/201 nt window, the distance to the closest neighboring input site at 2001/201 nt, and the distance to the closest neighboring A at 2001/201 nt; (vi) scores related to evolutionary conservation, such as the phastCons/fitCons scores of the nucleotide, and the average phastCons/fitCons scores within the flanking 101 nt; (vii) the attributes of the genes or transcripts, such as the miRNA targeted genes, miRNA targeted sites verified by experiments, overlapped binding regions of METTL3, and so on; (viii) genomic properties, including sncRNA, lncRNA, the number of isoforms/exons, housekeeping genes, the GC composition of genes, and the GC composition of 101 nt; (ix) SNP features [60], i.e., the distribution of the single-nucleotide polymorphism sites of the mRNA sequence around m^6^A sites. It is noteworthy that most of these features can be generated by the GenomicFeatures R package.

#### 2.4.4. Integrated Features

To further boost the prediction performance, many m^6^A site predictors [59,60,62,65] integrate multiple types of features, rather than only employing a single type of feature mentioned above. For example, by integrating context-, structure-, and genome-based features using SVM, WHISTLE [59] achieved promising prediction performance with an average AUROC of 0.948 and 0.880 on the independent test datasets from full transcripts and mature mRNA, respectively. SRAMP [63] used context- and structure-based features, including the predicted binary encoding, KNN encoding, spectrum encoding, and RNA loop region, to describe sample sequences and achieved high specificity.

#### 2.4.5. Feature Selection

Feature selection is an effective way to eliminate redundant features and reduce the large calculation burden and overfitting caused by high-dimensional vectors. Several filter-based feature selection methods have been adopted and proven to be effective in boosting the computational efficiency and prediction performance. There are three major types of filter-based feature selection methods: (i) the minimum redundancy maximum relevance (mRMR) [32,70], which is applied to calculate and rank the contribution values of each feature to achieve the optimal feature subset; (ii) elastic net (EN) [73], which is an approach that can implement feature selection based on regularized terms; (iii) statistical algorithms, such as the F-test [38], and F-score [57,72], which are employed to measure and rank the significance of all of the extracted feature components.

### 2.5. Predictive Algorithms Employed

There are three types of major prediction algorithms for m^6^A prediction (Supplementary Material Figure S1 and Table 1), namely (i) traditional machine learning-based methods, such as support vector machine (SVM) [26,27,28,29,30,31,32,37,38,40,42,43,45,46,47,48,59,62,64,65,70], random forest (RF) [33,63], XGBoost [34,57,60], AdaBoost [61], CatBoost [53], and second-order Markov models [51]; (ii) deep learning-based methods, such as deep neural networks (DNNs) [73], convolutional neural networks (CNNs) [39,41,49,52,54,71,74], and CNNs in combination with bidirectional long short-term memory (BiLSTM) [58,66,68]; and (iii) ensemble learning-based methods, such as machine learning-based ensemble methods [35,36,44,55] and ensemble deep learning [50,56,67,69,72].

#### 2.5.1. Traditional Machine Learning-Based Methods

Among the predictors analyzed in this study, SVM is the most widely used machine learning algorithm (Supplementary Material Figure S1B). When constructing these SVM-based m^6^A site predictors, the radial basis function (RBF) was generally considered as the kernel function, while a grid search strategy and K-fold cross-validation were employed to optimize the two parameters of SVM, i.e., the regularization parameter *C* and the kernel width parameter γ. However, it is time-consuming to perform this parameter optimization process, especially if the sample size is large. For large m^6^A datasets, the boosting method is an excellent choice, which generates strong learners by integrating weak learners based on the idea of ensemble learning. Five predictors were built using the boosting method: three for XGBoost [34,57,60], one for AdaBoost [61], and one for CatBoost [53]. All of them illustrated excellent prediction on their own datasets in comparison to other machine learning methods, such as SVM, RF, or KNN. At the same time, random forest [33], by integrating multiple decision trees, can reduce the risk of overfitting associated with a single model and improve the predictive accuracy, making it a good choice for datasets with high-dimensional features. Given the robust performance of SVM, RF, boost, and Markov models, we categorized these four classical models separately (Supplementary Material Figure S1B).

#### 2.5.2. Deep Learning-Based Methods

Deep learning techniques, advancing from traditional artificial neural network frameworks, have been widely applied in bioinformatics, such as in protein modification sites [99,100,101], gene ontology function prediction [102,103], medical image analysis [104], drug design [105,106,107], HLA epitope prediction [108,109,110], and so on. Among them, a typical feed-forward artificial neural network, the CNN, has attracted widespread attention and has been widely used in m^6^A site prediction. Many predictors [40,41,49,52,54,58,66,68,71,74] have been successively proposed using CNNs or CNNs in combination with other strategies (BiLSTM) based on single-nucleotide m^6^A data.

#### 2.5.3. Ensemble Learning-Based Methods

At the forefront of machine learning, ensemble learning, which integrates the output of multiple models, has exerted a significant impact on various field of bioinformatics as this technology can generally obtain better generalizability in comparison to a single model. Among the m^6^A predictors analyzed, in addition to homogeneous ensemble models—such as RF and XGBoost, which use decision trees as base learners—there are also heterogeneous ensemble methods based on different machine learning models [35,36,44,55], as well as heterogeneous ensemble methods combining various deep learning models [50,56,67,69,72]. For example, Zhang et al. constructed a machine learning-based ensemble model named M6A-GSMS [44], which integrated the output of six base classifiers, namely RF, extra trees (ET), bagging, SVM, Adaboost, and LightGBM, as the input of a meta-classifier, i.e., Gaussian Naïve Bayes (GaussianNB), to achieve the final prediction result. Chen et al. developed an ensemble deep learning framework, DeepM6Aseq-EL [69], consisting of five sub-networks, each of which was stacked by LSTM and a CNN.

### 2.6. Strategies and Measures for Performance Assessment

Three performance evaluation strategies, namely the jackknife validation test, K-fold cross-validation (CV), and independent test, are usually adopted to evaluate the prediction performance of the proposed predictors. The jackknife validation test, also termed leave-one-out CV, refers to a strategy where each sample is in turn used as test data to evaluate the predictor trained by the remaining data. K-fold CV is another similar strategy in which the dataset is randomly divided into k equal or almost equal subsets, and then each subset is in turn used as test data to evaluate the prediction performance of the predictor trained on the remaining subsets. Although the jackknife test is stricter and more objective, K-fold CV has more advantages in terms of the computing time, especially for large sample sizes. For this reason, most predictors surveyed in this study employ K-fold CV (k = 5 or 10) to construct performance matrices. Moreover, in order to compare them with existing methods fairly and objectively, an independent test is usually implemented using newly constructed data that have not been seen in the training data of the compared predictors.

In the field of bioinformatics [95,111,112,113,114,115,116,117], several widely used performance metrics have been employed to quantitatively measure the prediction performance, including the accuracy (Acc), sensitivity (Sn), specificity (Sp), Matthew’s correlation coefficient (MCC), F1-score, precision, area under the precision–recall curve (AUPR), and area under the receiver operating characteristic curve (AUROC) [110,118,119]. These metrics are formulated as follows:

(1) $$\left\{\begin{array}{c} \begin{array}{c} \begin{array}{c} \begin{array}{cc} Sn=\frac{TP}{TP+FN} & \;0\leq Sn\leq 1 \end{array}\\ \begin{array}{cc} Sp=\frac{TN}{TN+FP} & \;0\leq Sp\leq 1 \end{array} \end{array}\\ \begin{array}{cc} Precision=\frac{TP}{TP+FP} & 0\leq Precision\leq 1 \end{array}\\ \begin{array}{cc} F1=\frac{2TP}{2TP+FP+FN} & \;0\leq F1\leq 1 \end{array} \end{array}\\ \begin{array}{cc} Acc=\frac{TP+TN}{TP+TN+FP+FN} & \;0\leq Acc\leq 1 \end{array}\\ \begin{array}{cc} MCC=\frac{TP\times TN-FP\times FN}{\sqrt{\left(TP+FN\right)\times \left(TN+FN\right)\times \left(TP+FP\right)\times \left(TN+FP\right)}} & -1\leq MCC\leq 1 \end{array} \end{array}\right.\;$$

where *TP*, *FP*, *TN*, and *FN* stand for the numbers of true positives, false positives, true negatives, and false negatives, respectively.

### 2.7. Webserver/Software Availability and Usability

A user-friendly webserver and/or local software can significantly facilitate scholars’ implementation of m^6^A prediction using the proposed computational methods. In total, 67.3% (35 out of 52) of the m^6^A site predictors surveyed in this study have available online websites or stand-alone software, and 60.0% (21 out of 35) of them are still active (Table 1).

The query sequences in the FASTA format are allowed to be submitted to the above-mentioned webservers for the implementation of m^6^A site prediction online. However, some servers limit the length and number of the submitted sequences. For example, TS-m6A-DL only allows a fixed length of 41 nt for each sequence, while m6A-NeuralTool was designed to analyze fixed 101-nt-long sequences from *A. thaliana*, 51 nt from *S. cerevisiae*, and 41 nt from *M. musculus/H. sapiens*. iRNA(m6A)-PseDNC allows query sequences with a minimum length of 51 nt to be submitted, while they must be no shorter than 41 nt for iRNA-PseColl, iRNA-3typeA, iRNA-m6A, and MethyRNA; M6ATH requires no more than 100 sequences for each submission.

In addition, it is critical for users that the output format of webservers is well designed to facilitate the understanding and interpretation of the prediction results. All of the available servers surveyed in this study can offer immediate prediction results on their webpages after submitting the query sequences. iRNA-3typeA, m6A-NeuralTool, and TS-m6A-DL can provide the output containing the predicted labels (e.g., m^6^A or non-m^6^A sequences). Most methods, such as iRNA-Methyl, m6Apred, iRNA(m6A)-PseDNC, iRNA-PseColl, MethyRNA, and SRAMP, offer the probability scores and predicted positions of m^6^A or non-m^6^A as well.

## 3. Experimental Results

### 3.1. Performance Comparison of Species-Specific Predictors

**Figure 2:**
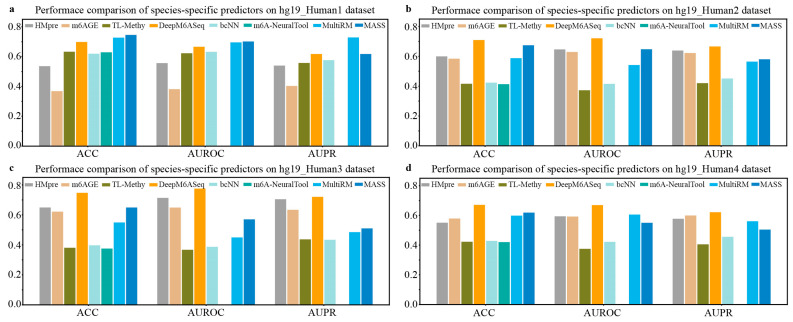
Performance comparison of species-specific predictors on independent datasets of *H. sapiens*.

We collected m^6^A site sequences for nine species, as depicted in Table 3, and conducted a series of experiments to evaluate the performance of species-specific predictors. First, we utilized independent m^6^A data from four different sources of H. sapiens to evaluate the generalization performance of the existing species-specific predictors available, including DeepM6ASeq, HMpre, bCNN, m6AGE, m6A-NeuralTool, MultiRM, TL-Methy, and MASS. The results, shown in Figure 2, indicate that the predictor DeepM6ASeq demonstrates outstanding predictive performance on independent datasets from different sources. On the hg19_Human2 dataset, DeepM6ASeq achieves an ACC of 70.92% and an AUROC of 0.7211, surpassing the second-best predictor, MASS, by 3.5% and 7.36%, respectively. Additionally, its AUPR is 0.6663, exceeding the second-best predictor, m6AGE, by 4.41%. On the hg19_Human3 and hg9_Human4 datasets, the ACC, AUROC, and AUPR of DeepM6ASeq are all higher than those of the other predictors, while its performance on the hg19_Human1 dataset is also comparable to that of the other predictors. Furthermore, as seen from Figure 2, MultiRM and MASS also exhibit stable predictive performance in predicting the m^6^A sites of H. sapiens.

**Figure 3:**
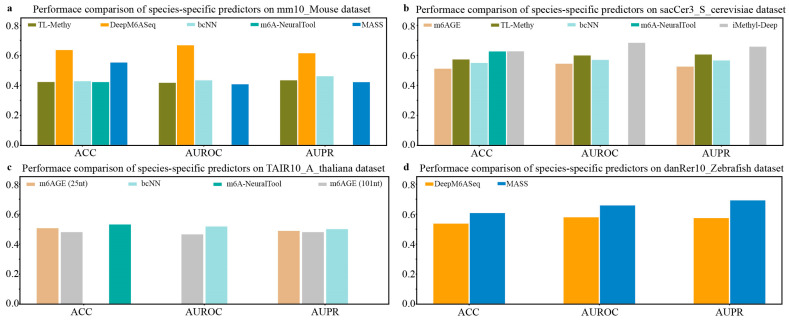
Performance comparison of species-specific predictors on independent datasets of *M. musculus*, *S. cerevisiae*, *A. thaliana*, and *Zebrafish.*

Next, we compared the prediction performance of species-specific predictors on independent datasets of *M. musculus*, *S. cerevisiae*, *A. thaliana*, and *Zebrafish*. As shown in Figure 3a, among the available species-specific predictors for the prediction of the m^6^A sites of *M. musculus*, DeepM6ASeq achieves the best predictive performance, followed by MASS. It can be seen from Figure 3b that iMethy-Deep demonstrates the most superior performance for m^6^A site prediction on the independent dataset sacCer3_S_cerevisiae of *S. cerevisiae.* The results depicted in Figure 3c suggest that the available species-specific predictors, such as m6AGE (25nt), m6AGE (101nt), bCNN, and m6A-NeuralTool, exhibit comparable prediction performance in predicting the m^6^A sites of A. thaliana. Additionally, on the independent dataset danRer10_Zebrafish, the species-specific predictor MASS exhibits slightly higher values for the ACC, AUROC, and AUPR compared to another predictor, DeepM6ASeq (Figure 3d).

Overall, species-specific deep learning-based methods, such as DeepM6ASeq, MultiRM, and MASS, demonstrate superior predictive performance compared to traditional machine learning methods, such as HMpre, m6AGE, and TL-Methy. In particular, among them, DeepM6ASeq and MASS can predict m^6^A sites across multiple species.

### 3.2. Cross-Species Validation of State-of-the-Art Predictors

**Figure 4:**
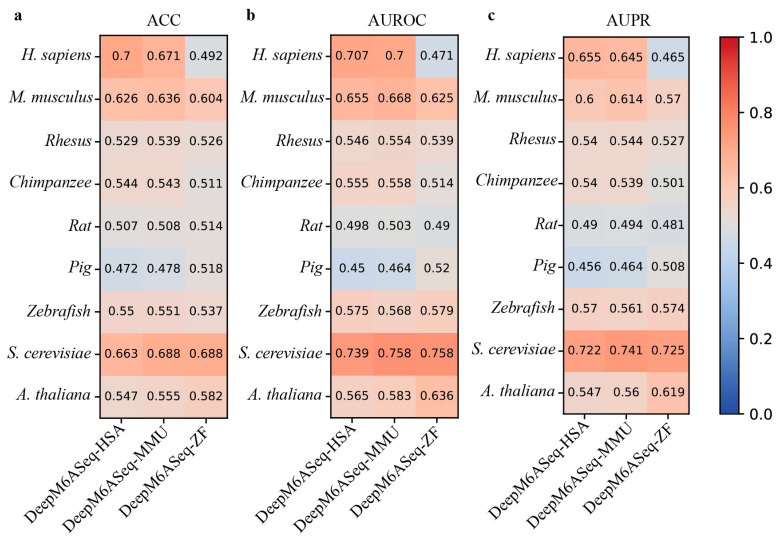
Cross-species performance comparison of DeepM6ASeq predictor on independent datasets of 9 species. (**a**–**c**) respectively show the values for ACC, AUROC, and AUPR.

Considering that the performance of DeepM6ASeq and MASS is superior to that of the current methods, we next evaluate the cross-species prediction performance of these predictors on the independent datasets of nine species. DeepM6ASeq consists of three sub-models, DeepM6ASeq-HSA, DeepM6ASeq-MMU, and DeepM6ASeq-ZF, trained on m^6^A site data from *H. sapiens*, *M. musculus*, and *Zebrafish*, respectively. Thus, we first merge the four *H. sapiens* datasets, namely hg19_Human1, hg19_Human2, hg19_Human3, and hg19_Human4, into a larger dataset and then test the cross-species prediction performance of these sub-models of DeepM6ASeq on nine independent datasets. The results depicted in Figure 4 indicate that when species-specific predictors are used to predict cross-species m^6^A sites, their predictive performance shows a significant decrease. For example, DeepM6ASeq-HSA achieves an ACC of 0.7 on the *H. sapiens* datasets, while its ACC drops to 0.626, 0.529, 0.544, 0.507, 0.472, 0.55, 0.663, and 0.547 on the other cross-species datasets, respectively.

Although DeepM6ASeq-HSA performs well on the *H. sapiens* dataset, its performance declines on the cross-species datasets. Despite some genes being conserved across multiple species, significant differences in gene expression regulation and biological functions may exist between species, leading to the model’s inability to effectively generalize from *H. sapiens* datasets to other species. In contrast, the DeepM6ASeq-MMU model achieved the top ACC values of 0.671 and 0.688 on the *H. sapiens* and *S. cerevisiae* datasets, respectively, and the DeepM6ASeq-ZF model achieved the top ACC values of 0.604 and 0.688 on the *M. musculus* and *S. cerevisiae* datasets, demonstrating their good generalization abilities. This indicates that both models can effectively capture and utilize shared sequence information among different species, thereby improving the prediction accuracy.

**Figure 5:**
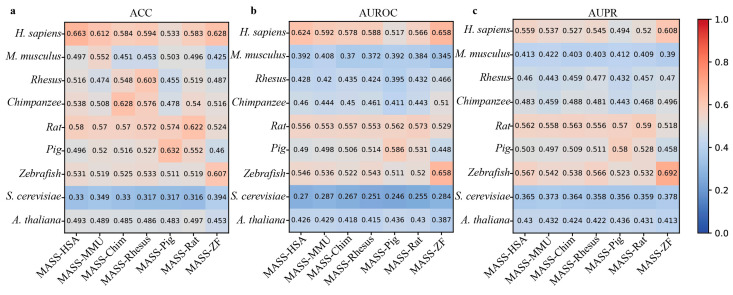
Cross-species performance comparison of MASS predictor on independent datasets of 9 species. (**a**–**c**) respectively show the values for ACC, AUROC, and AUPR.

In addition, MASS contains seven species-specific sub-models. Therefore, we next use the independent datasets derived from nine species to evaluate the cross-species prediction performance of these sub-models. From Figure 5, it can be observed that these species-specific sub-models exhibit varying degrees of performance decline when predicting m^6^A sites in different species. For example, the MASS-HSA predictor trained on human m^6^A data achieves an ACC of 0.663 when predicting human m^6^A sites, while the accuracy on the independent datasets of the other eight species is 0.497, 0.516, 0.538, 0.58, 0.486, 0.531, 0.33, and 0.493, respectively.

Furthermore, to make our evaluation more comprehensive, we also assessed the cross-species prediction performance of seven other predictors—MultiRM, TL-Methy, m6AGE, iMethyl-Deep, HMpre, bCNN, and m6A-NeuralTool—and present the results in Supplementary Material S2. Similar to the findings for DeepM6ASeq and MASS, the performance metrics of these predictors across different species, such as the ACC, AUROC, and AUPR, also indicate that species-specific predictors perform better when predicting m^6^A sites within their own species, while their performance often declines in cross-species predictions. For example, MultiRM and iMethyl-Deep exhibit high prediction performance on their training species but show significantly reduced performance on other species. This further suggests that constructing species-specific prediction models is necessary for the accurate identification of m^6^A sites.

### 3.3. Performance Comparison for Prediction of m^6^A Sites in Single Cells

The current computational methods for m^6^A site prediction are all based on bulk-cell sequencing data, while the emerging scDART-seq technology can achieve the identification of single-nucleotide m^6^A sites at a transcriptome-wide level in single cells. Therefore, we need to evaluate whether the current methods can accurately predict m^6^A sites at the single-cell level.

**Figure 6:**
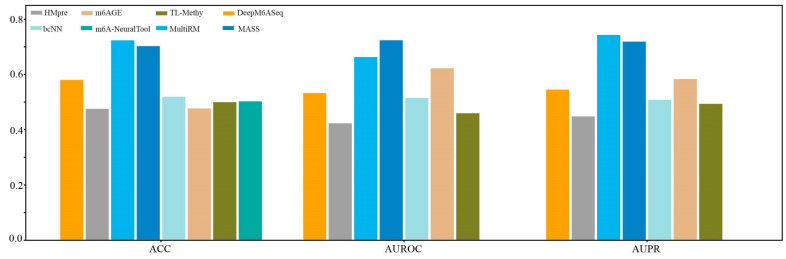
Comparison of 8 predictors on the *H. sapiens* m^6^A single-cell dataset.

We first constructed a dataset, named Hg38_Human, which contained 11124 single-cell m^6^A site sequences from scDART-seq data and 11124 non-m^6^A site sequences. Subsequently, we compared the prediction performance of the currently available species-specific predictors in identifying m^6^A sites in *H. sapiens* at a single-cell level on the independent dataset Hg38_Human. As shown in Figure 6, MultiRM achieves an ACC of 0.7233, an AUROC of 0.6628, and an AUPR of 0.7428, while MASS achieves an ACC of 0.7025, an AUROC of 0.7236, and an AUPR of 0.7118. Compared with other prediction methods, MultiRM has the best prediction performance in predicting m^6^A sites in single cells, followed by MASS. Furthermore, among these predictors, the predictive performance of deep learning-based methods, including MultiRM, MASS, DeepM6ASeq, bCNN, and m6A-NeuralTool, is significantly better than that of traditional machine learning methods, such as HMpre, m6AGE, and TL-Methy.

## 4. Conclusions

In this study, we have surveyed 52 state-of-the-art computational approaches for the prediction of m^6^A sequences and benchmarked nine species-specific predictors with available and functioning webservers/local tools. To the best of our knowledge, this study represents the most comprehensive and large-scale benchmarking test of predictors for the identification of m^6^A sites. A wide range of aspects were summarized in detail, including the employed algorithms, calculated features, performance evaluation strategies, and software usability. By curating 13 independent test datasets for various species, we extensively benchmarked nine available predictors and demonstrated their prediction performance, including species-specific prediction, cross-species prediction, and the identification of m^6^A sites in single cells. The prediction results show that deep learning-based methods generally outperform traditional machine learning-based methods. Although the performance of the surveyed predictors is subject to change in cases where different test datasets and new data are used to test the models, DeepM6ASeq, MultiRM, and MASS have the best generalization performance. We expect this analysis to be a stepping stone toward the design and implementation of more accurate predictors for m^6^A sites in the future.

## Figures and Tables

**Table 1:** Detailed information of existing models.

Tool	Species	Experimental Method	Sequence Length (nt)	Features ^a^	Algorithm ^d^	Evaluation Strategy	Year	Webserver ^b^	Data Size ^c^
iRNA-Methyl [26]	*SC*	m^6^A-Seq	51	PseDNC	SVM	Jackknife	2015	http://lin-group.cn/server/iRNA-Methyl (accessed on 12 September 2022)	Smet1307
m6Apred [37]	*SC*	m^6^A-Seq	21	CPD	SVM	Jackknife independent test	2015	http://lin-group.cn/server/m6Apred (accessed on 12 September 2022)	Smet1307sub
pRNAm-PC [27]	*SC*	m^6^A-Seq	51	PseDNC, AC, CC	SVM	Jackknife	2016	Decommissioned	Smet1307
RNA-MethylPred [28]	*SC*	m^6^A-Seq	51	DNC, KNN scores	SVM	Jackknife	2016	No	Smet1307
M6A-HPCS [29]	*SC*	m^6^A-Seq	51	HPCS	SVM	10-fold CV	2016	Decommissioned	Smet1307
TargetM6A [38]	*SC*	m^6^A-Seq	21	PSNP, PSDP, NC	SVM	Jackknife, independent test	2016	Decommissioned	Smet1307sub
RAM-ESVM [35]	*SC*	m^6^A-Seq	51	PseDNC	Ensemble SVM	10-fold CV	2017	Decommissioned	Smet1307
iRNA(m6A)-PseDNC [30]	*SC*	m^6^A-Seq	51	PseDNC	SVM	10-fold CV	2018	http://lin-group.cn/server/iRNA(m6A)-PseDNC.php (accessed on 12 September 2022)	Smet1307
M6APred-EL [36]	*SC*	m^6^A-Seq	51	PS(k-mer)NP, RFHC-GACs, AC, CC	Ensemble (SVM)	10-fold CV	2018	Decommissioned	Smet1307
DeepM6APred [31]	*SC*	m^6^A-Seq	51	Deep features, NPPS	SVM	10-fold CV	2018	Decommissioned	Smet1307
M6A-PXGB [34]	*SC*	m^6^A-Seq	51	PSNP, PSDP, NC	XGBoost	10-fold CV	2019	No	Smet1307
m6A-pred [33]	*SC*	m^6^A-Seq	51	CPD, DNC, TNC	RF	10-fold CV	2020	No	Smet1307
m6A-Finder [32]	*SC*	m^6^A-Seq	51	AC, NC	SVM	Jackknife	2022	No	Smet1307
iMethyl-Deep [39]	*SC*	m^6^A-Seq, m^6^A-CLIP, miCLIP	51	One-hot	CNN	10-fold CV, independent test	2020	https://github.com/abdul-bioinfo/iMethyl-deep (accessed on 12 September 2022)	Smet1307; Smet3270
iMethyl-STTNC [45]	*SC*, *HSA*	m^6^A-Seq	51-41	PseDNC, PseTNC, STNC, STTNC	SVM	10-fold CV	2018	No	Smet1307; Hmet1130
iRNA-PseColl [40]	*HSA*	m^6^A-Seq	41	CPD	SVM	Jackknife	2017	http://lin-group.cn/server/iRNA-PseColl.html (accessed on 12 September 2022)	Hmet1130
iRNA-Mod-CNN [41]	*HSA*	m^6^A-Seq	41	K-Gram	CNN	5-fold CV	2021	No	Hmet1130
HMpre [60]	*HSA*	miCLIP	51	SLRF, FREI, SNP	XGBoost	Independent test	2018	No	26,512:271,214
WHISTLE [59]	*HSA*	m^6^A-CLIP, miCLIP	unknown	CPD, Genomic features	SVM	5-fold CV, independent test	2019	No	37,899 (1:10)
m6Aboost [61]	*HSA*	miCLIP	21	Experimental and sequence features	AdaBoost	5-fold CV, independent test	2021	No	11,701:42,090
MultiRM [58]	*HSA*	m^6^A-CLIP, miCLIP	51	One-hot	CNN+BiLSTM	Independent test	2021	Decommissioned	65,178 (1:1)
DeepM6ASeq-EL [69]	*HSA*	m^6^A-CLIP, miCLIP	unknown	One-hot, CPD, Word2vec	Ensemble (CNN+LSTM)	Independent test	2022	No	37,899 (1:10)
ConsRM [65]	*HSA*	m^6^A-CLIP, m^6^A-REF-Seq, miCLIP	11	CPD, Genomic features	SVM	5-fold CV, independent test	2021	http://180.208.58.19/conservation/ (accessed on 12 September 2022)	177,998 (1:1)
MethyRNA [46]	*HSA*, *MMU*	m^6^A-Seq, MeRIP-Seq	41	CPD	SVM	Jackknife	2016	http://lin-group.cn/server/MethyRNA (accessed on 12 September 2022)	Hmet1130 Mmet725
SRAMP [63]	*HSA*, *MMU*	miCLIP	W	One-hot, SPE, KNN scores, PSSP	RF	5-fold CV, independent test	2016	http://www.cuilab.cn/sramp/ (accessed on 12 September 2022)	57,433, mRNA; 68,083, full transcripts (1:10)
RNAMethPre [62]	*HSA*, *MMU*	MiCLIP-seq, m^6^A-CLIP	101	One-hot, NC, SLS	SVM	5-fold CV, independent test	2016	Decommissioned	*HSA:* 29,547, mRNA; 31,728, full transcripts (1:1) *MMU*: 22,740, mRNA; 24,705, full transcripts (1:1)
iRNA-3typeA [47]	*HSA*, *MMU*	m^6^A-Seq, MeRIP-Seq	41	CPD	SVM	Jackknife	2018	http://lin-group.cn/server/iRNA-3typeA.php (accessed on 12 September 2022)	Hmet1130; Mmet725
Gene2vec [67]	*HSA*, *MMU*	MiCLIP-seq, m^6^A-CLIP	1001	One-hot, NMSE, word embedding	CNN+ensemble	10-fold CV, independent test	2019	Decommissioned	495,572 (1:10)
M6ATH [42]	*At*	m^6^A-seq	25	CPD	SVM	Jackknife	2016	http://lin-group.cn/server/M6ATH (accessed on 12 September 2022)	Amet394
AthMethPre [43]	*At*	m^6^A-seq, MeRIP-seq	41	One-hot, PIkmer	SVM	5-fold CV, independent test	2016	Decommissioned	5081 (1:1)
RAM-NPPS [48]	*SC*, *HSA*, *At*	m^6^A-Seq, PA-m^6^A-seq	51	NPPS	SVM	10-fold CV	2017	Decommissioned	Smet1307; Hmet8366; Amet394
m6A-word2vec [49]	*SC*, *HSA*, *At*	m^6^A-Seq, PA-m^6^A-seq	51	Word embedding	CNN	10-fold CV	2020	No	Smet1307; Hmet1130; Amet394
m6AGE [53]	*SC*, *HSA*, *At*	m^6^A-Seq, PA-m^6^A-seq	21-41-25-101	Graph embedding, sequence-derived features (CTD, PseKNC, NPS, NPPS, CPD, EIIP, BPB)	CatBoost	5-fold CV	2021	https://github.com/bokunoBike/m6AGE (accessed on 12 September 2022)	Smet1307; Hmet1130; Amet394; Amet2518
DeepM6ASeq [66]	*HSA*, *Mouse*, *ZF*	miCLIP-Seq	101	One-hot	CNN+BiLSTM	5-fold CV, independent test	2018	https://github.com/rreybeyb/DeepM6ASeq (accessed on 12 September 2022)	*HSA*: 49,050; *Mouse*: 37,716; *ZF*: 22,108 (1:1)
iN6-Methyl (5-step) [50]	*SC*, *HSA*, *MMU*	m^6^A-seq, MeRIP-seq	51-41-41	Word embedding	CNN	10-fold CV	2019	decommissioned	Smet1307; Hmet1130; Mmet725
Chong et al. [52]	*SC*, *HSA*, *MMU*	m^6^A-seq, MeRIP-seq	51-41-41	k-mer	CNN	10-fold CV	2021	No	Smet1307; Hmet1130; Mmet725
MM-m6APred [51]	*SC*, *HSA*, *MMU*	m^6^A-seq, MeRIP-seq	51-41-41	Probability matrix	Second-order Markov	10-fold CV	2021	decommissioned	Smet1307; Hmet1130; Mmet725
M6AMRFS [57]	*SC*, *HSA*, *MMU*, *At*	m^6^A-seq, MeRIP-seq	51-41-41-101	One-hot; LPSDF	XGBoost	5-fold CV, independent test	2018	decommissioned	Smet1307; Hmet1130; Mmet725; Amet2100
bCNN-Methylpred [54]	*SC*, *HSA*, *MMU*, *At*	m^6^A-seq, MeRIP-seq, miCLIP-seq	51-41-41-101	Circular encoding, one-hot, NCP	CNN	10-fold CV	2021	https://github.com/Naeem-jbnu/RNA_Modification_Sites (accessed on 12 September 2022)	Smet1307; Hmet1130; Mmet725; Amet1000
m6A-NeuralTool [56]	*SC*, *HSA*, *MMU*, *At*	m^6^A-seq, MeRIP-seq	51-41-41-101	One-hot	Ensemble (CNN, SVM, NB)	10-fold CV, independent test	2021	http://nsclbio.jbnu.ac.kr/tools/m6A-NeuralTool/ (accessed on 12 September 2022)	Smet3270; Hmet1130; Mmet725; Amet2100
TL-Methy [55]	*SC*, *HSA*, *MMU*, *Rice*	m^6^A-seq, MeRIP-seq	51-41-41-41	NAC, DNC, TNC, PSTNP, BPB, one-hot, CPD	Ensemble (SVM, KNN, LR, DA)	10-fold CV	2022	https://github.com/LDWang-dlmu/N6-methyladenine (accessed on 12 September 2022)	Smet1307; Hmet1130; Mmet725; Rmet880
M6A-BiNP [64]	*SC*, *HSA*, *MMU*, *Rat*, *At*	m^6^A-seq, MeRIP-seq, miCLIP-seq, m^6^A-REF-seq	51-41-41-41-25	PSP-PMI, PSP-PJMI	SVM	10-fold CV	2021	https://github.com/Mingzhao2017/M6A-BiNP (accessed on 12 September 2022)	Smet1307; Hmet1130; Mmet725; Amet394; Species-/tissue-specific datasets; Human51 (1:1)
M6A-GSMS [44]	*SC*, *HSA*, *MMU*, *At*, *DM*	m^6^A-seq, MeRIP-seq	51-41-41-101-41	NMBAC, PC-PseDNC-General, PseDPC, one-hot, K-mer	Ensemble (RF, ET, SVM, LGBM, Bagging, Adaboost)	10-fold CV	2021	https://github.com/Wang-Jinyue/M6A-GSMS (accessed on 12 September 2022)	Smet1307; *HSA*: 5100; *MMU:* 725; *At*: 2100; DM: 300 (1:1)
MASS [68]	*HSA*, *MMU*, *Chim*, *Rhesus*, *Pig*, *Rat*, *ZF*	m^6^A-Seq, MeRIP-Seq, m^6^A-CLIP, miCLIP-seq	101	One-hot, phylogenetic tree	CNN+BiLSTM	5-fold CV	2021	https://github.com/mlcb-thu/MASS (accessed on 12 September 2022)	*HSA:* 305,644; *MMU*: 317,702; *Chim:* 26,248; *Rhesus:* 27,059; *Pig:* 81,501; *Rat:* 41,735; *ZF:* 19,834 (1:10)
iRNA-m6A [70]	*HSA*, *MMU*, *Rat*	m^6^A-REF-seq	41	AC, CC, CPD, one-hot	SVM	5-fold CV, independent test	2020	http://lin-group.cn/server/iRNA-m6A/ (accessed on 12 September 2022)	TSdata
im6A-TS-CNN [71]	*HSA*, *MMU*, *Rat*	m^6^A-REF-seq	41	One-hot	CNN	5-fold CV, independent test	2020	No	TSdata
Jia et al. [72]	*HSA*, *MMU*, *Rat*	m^6^A-REF-seq	41	One-hot, sequence feature, KNFR	Ensemble (CNN+capsule+BiGRU)	5-fold CV, independent test	2022	No	TSdata
DNN-m6A [73]	*HSA*, *MMU*, *Rat*	m^6^A-REF-seq	41	One-hot, TNC, ENAC, KSNPFs, CPD, PseDNC, PSNP, PSDP	DNN	5-fold CV, independent test	2021	https://github.com/GD818/DNN-m6A (accessed on 12 September 2022)	TSdata
TS-m6A-DL [74]	*HSA*, *MMU*, *Rat*	m^6^A-REF-seq	41	One-hot	CNN	5-fold CV, independent test	2021	http://nsclbio.jbnu.ac.kr/tools/TS-m6A-DL/ (accessed on 12 September 2022)	TSdata
Deepm6A-MT [80]	*HSA*, *MMU*, *Rat*	m^6^A-REF-seq	41	Word embedding, one-hot, NCP, CPD	CNN+BiGRU	5-fold CV, independent test	2024	http://www.biolscience.cn/Deepm6A-MT/ (accessed on 12 September 2022)	TSdata
MTTLm6A [81]	*SC*	m^6^A-CLIP, miCLIP-seq	601	One-hot	CNN	5-fold CV, independent test	2023	http://47.242.23.141/MTTLm6A/index.php (accessed on 12 September 2022)	49,338 (1:1)
m6A-TCPred [82]	*HSA*	PA-m^6^A-seq, miCLIP, m^6^A-REF-seq	Nucleotide position	NCP, EIIP	SVM	5-fold CV, independent test	2024	www.rnamd.org/m6ATCPred (accessed on 12 September 2022)	10,424:54,949

^a^ PseDNC: pseudo dinucleotide composition; DNC: dinucleotide composition; AC: auto-covariance; CC: cross-covariance; HPCS: heuristic nucleotide physical–chemical property selection; KNN scores: K-nearest neighbor encoding; PIkmer: position-independent k-mer frequency; PSNP: position-specific nucleotide propensity; PSDP: position-specific dinucleotide propensity; NC: nucleotide composition; PS(k-mer)NP: position-specific k-mer nucleotide propensity; NCP: nucleotide chemical property; CPD: chemical property with density; RFHC-GAC: a method integrating CPD, AC, and CC; NPPS: nucleotide pair position specificity; PseTNC: pseudo-trinucleotide composition; STNC: split-trinucleotide composition; STTNC: split-tetranucleotide composition; PSSP: predicted secondary structure pattern; spectrum encoding: nucleotide pair spectrum encoding; SLS: stability of the local structure; one-hot: binary encoding; SPE: spectrum encoding; SLRF: site location-related features; FREI: features related to entropy information; SNP: single-nucleotide polymorphism features; LPSDF: local position-specific dinucleotide frequency; MMSE: neighboring methylation state encoding; KSNPFs: K-spaced nucleotide pair frequencies; EIIP: electron–ion interaction pseudopotential. ^b^ Decommissioned—the webserver/tool is no longer available; no—the publication has no webserver or tool. ^c^ Smet1307: a dataset of m^6^A sites in the *S. cerevisiae* genome, consisting of 1307 positive samples and 1307 negative samples with 51 nucleotides; Smet1307sub: a subset of Smet1307, in which the 832 m^6^A sites with distances to the detected m^6^A-seq peaks less than 10 bp were selected as positive samples; Hmet1130: a dataset of m^6^A sites in the *H. sapiens* genome, consisting of 1130 positive samples and 1130 negative samples with 41 nucleotides. ^d^ SVM: a supervised learning algorithm that finds the hyperplane that best separates different classes in a dataset; XGBoost: an efficient, scalable implementation of gradient boosting that builds strong learners by combining multiple weak learners, typically decision trees; RF: an ensemble learning method that creates multiple decision trees from random subsets of the data to improve the predictive accuracy and reduce overfitting; CNN: a deep learning architecture particularly effective for image and spatial data processing due to its convolutional layers that capture local patterns; AdaBoost: an ensemble technique that iteratively improves weak classifiers by adjusting weights on misclassified samples to boost overall performance; BiLSTM: a recurrent neural network (RNN) variant that captures long-term dependencies in both forward and backward directions for sequence data; CatBoost: a gradient boosting algorithm optimized for categorical features, reducing overfitting and improving the prediction speed and accuracy; Markov Model: a probabilistic model that predicts future states based on the assumption that the current state is only dependent on the previous state; LGBM: a gradient boosting framework that uses a histogram-based algorithm for faster training and reduced memory consumption; BiGRU: a variant of GRU (a simplified LSTM) that processes sequence data in both directions to capture temporal dependencies.

## Data Availability

The original contributions presented in the study are included in the article/Supplementary Material, further inquiries can be directed to the corresponding authors.

## Supplementary Materials

The following supporting information can be downloaded at: https://www.mdpi.com/article/10.3390/biology13100777/s1. All 13 independent test datasets used for the benchmarking of various species-specific predictors in this study are available at “https://github.com/ztLuo-bioinfo/m6A/ (accessed on 20 September 2024)”.
