# Supplementary material for: Comprehensive Review and Assessment of Computational Methods for Prediction of N6-Methyladenosine Sites
Source: Biology (Basel). 2024 Sep 28;13(10):777. doi: 10.3390/biology13100777 (PMC11504118; doi:10.3390/biology13100777)
Supplement: Supplementary file 1 [file biology-13-00777-s001.zip › biology-3209320-supplementary.pdf]

Supplementary Material

Supplementary Material 1: Supplementary Figures S1 summarizes the progress of computational tools for predicting RNA N6-methyladenosine (m6A) sites.

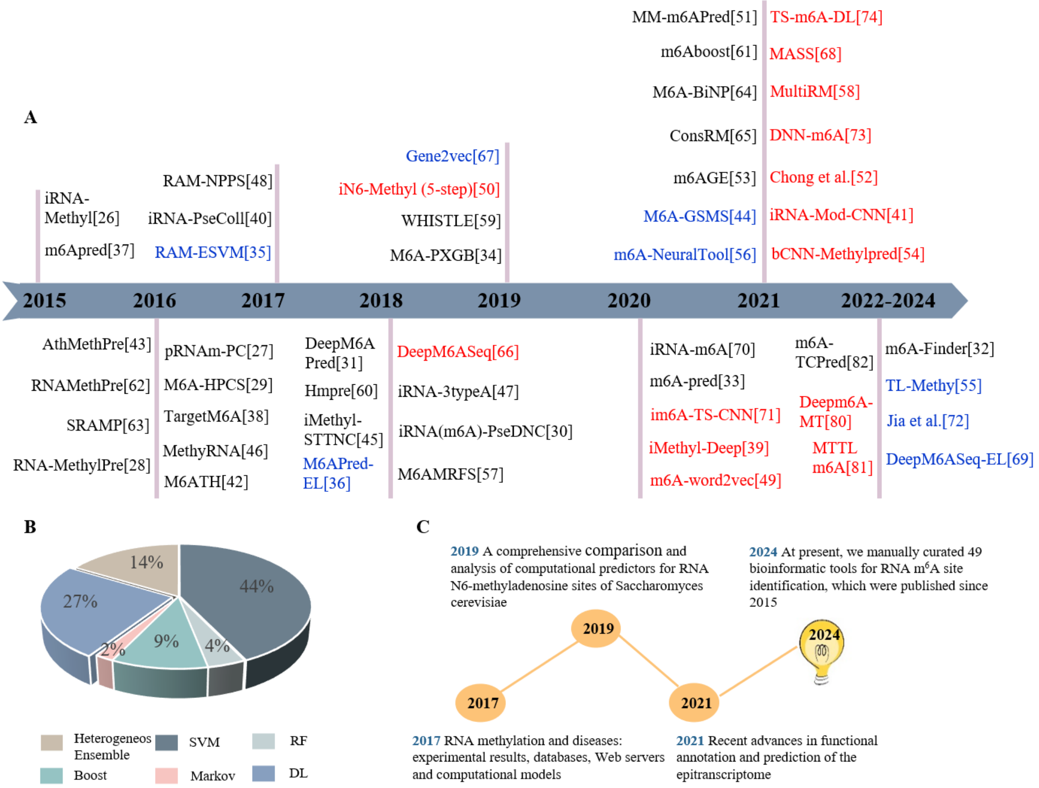

Figure S1. A timeline of (a) the details of computational approaches for predicting m6A sites; (b) Pie charts demonstrating the proportion of predictor types; and (c) historical reviews and assessments of these methods.

Supplementary Material 2: Supplementary Figures S2-8 display the cross-species prediction performance of the other seven predictors, including MultiRM, iMethyl-Deep, TL-Methy, m6AGE, HMpre, bCNN, and m6A-NeuralTool.

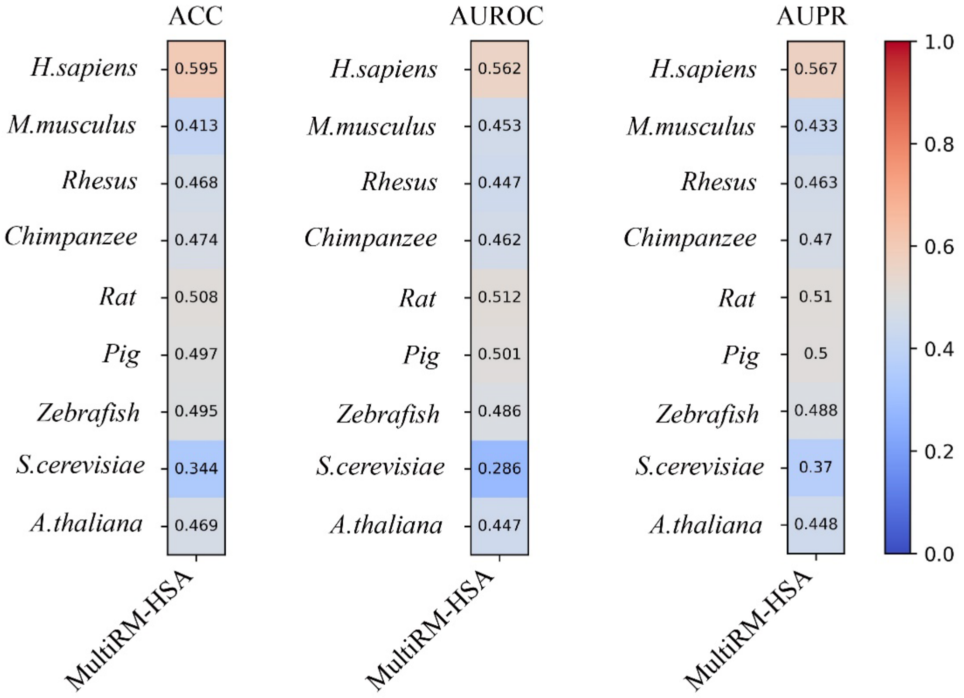

Figure S2. Cross-Species performance comparison of MultiRM predictor on independent datasets of 9 species.

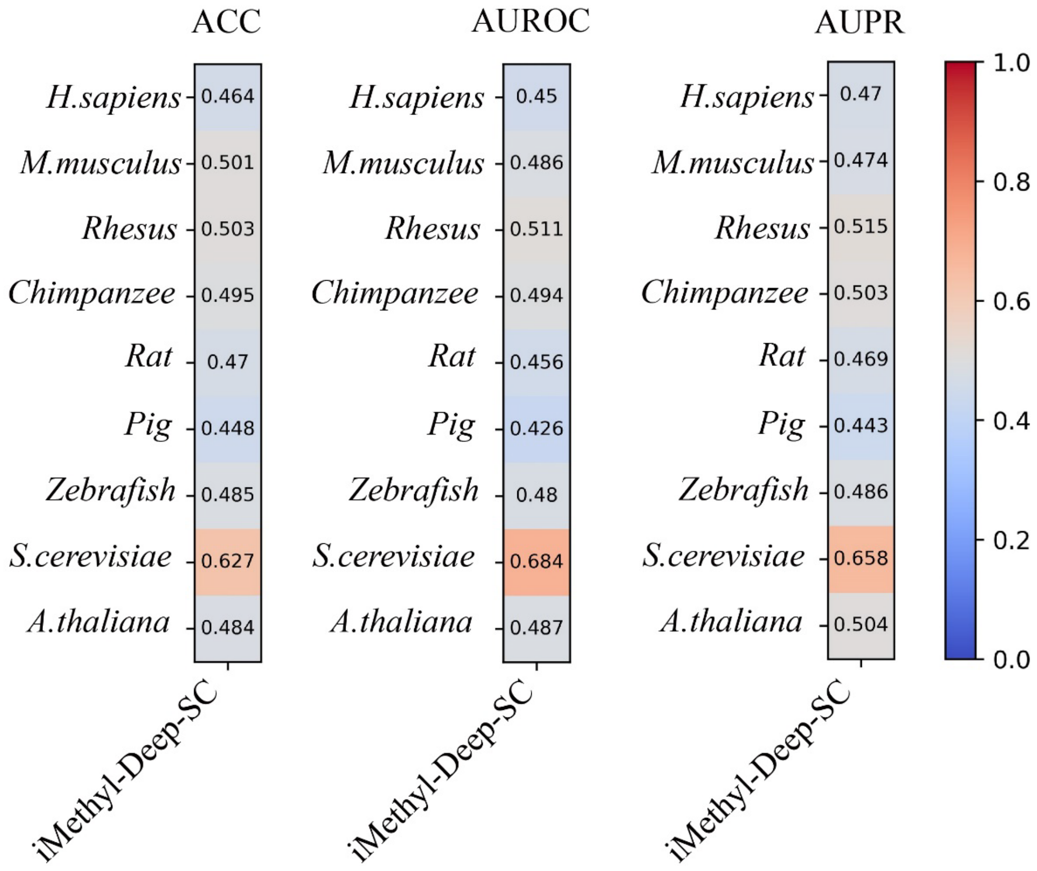

Figure S3. Cross-Species performance comparison of iMethyl-Deep predictor on independent datasets of 9 species.

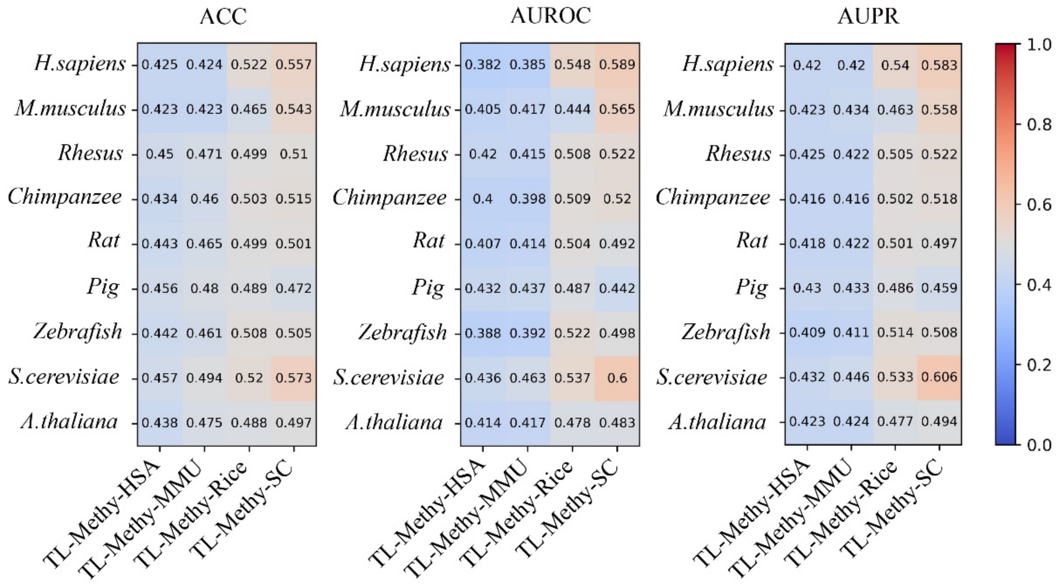

Figure S4. Cross-Species performance comparison of TL-Methy predictor on independent datasets of 9 species.

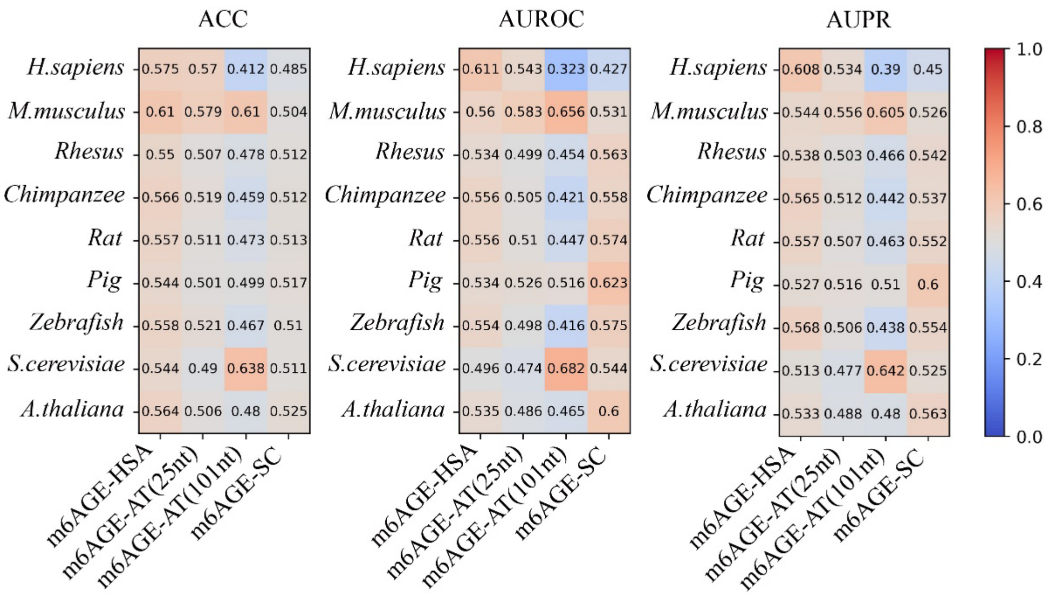

Figure S5. Cross-Species performance comparison of m6AGE predictor on independent datasets of 9 species.

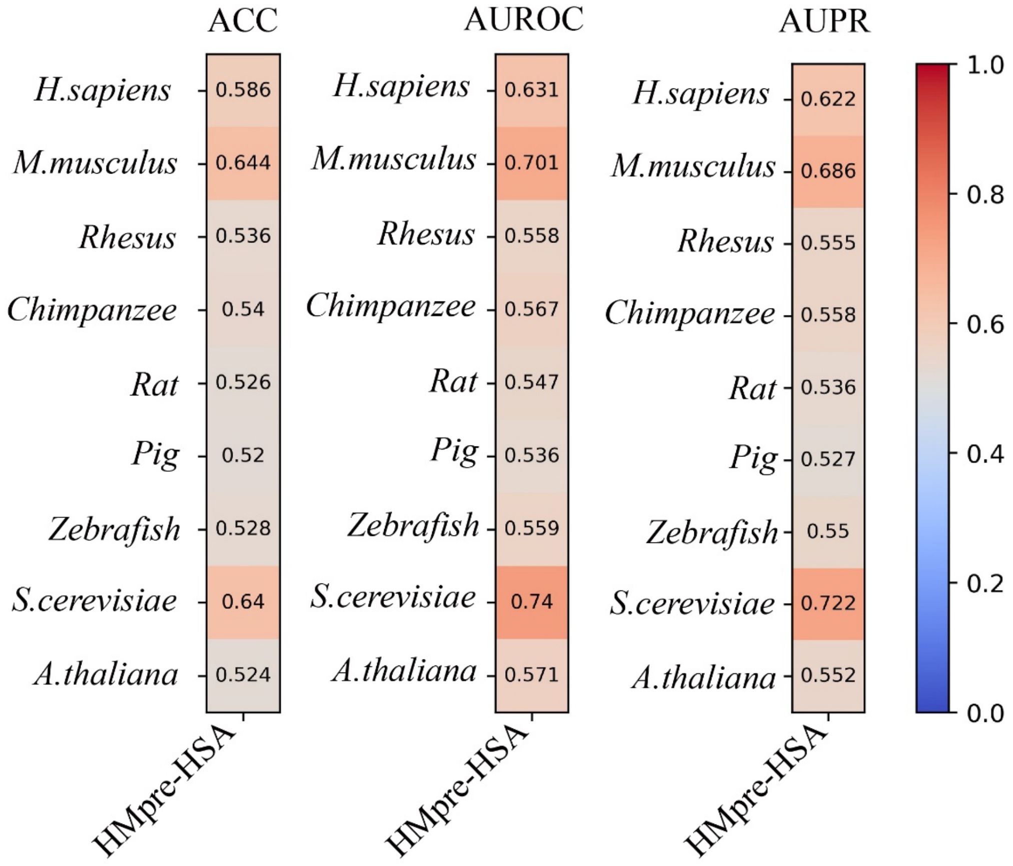

Figure S6. Cross-Species performance comparison of HMpre predictor on independent datasets of 9 species.

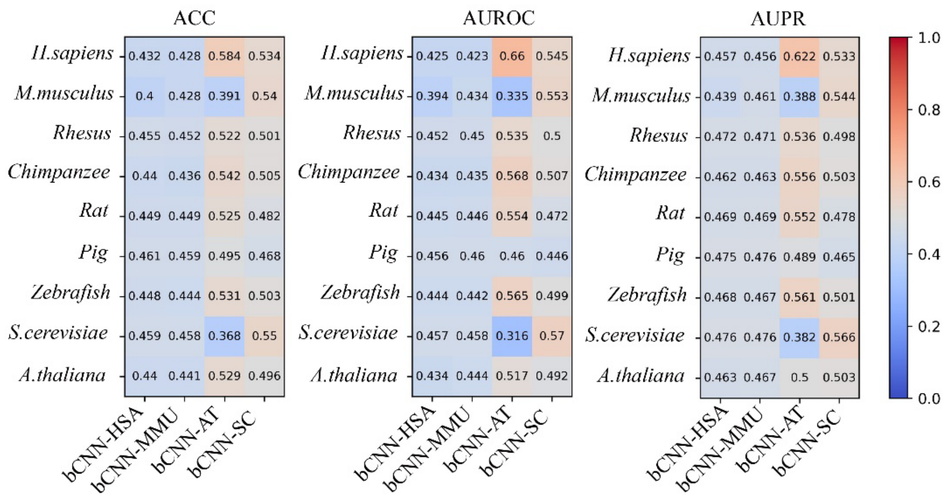

Figure S7. Cross-Species performance comparison of bCNN predictor on independent datasets of 9 species.

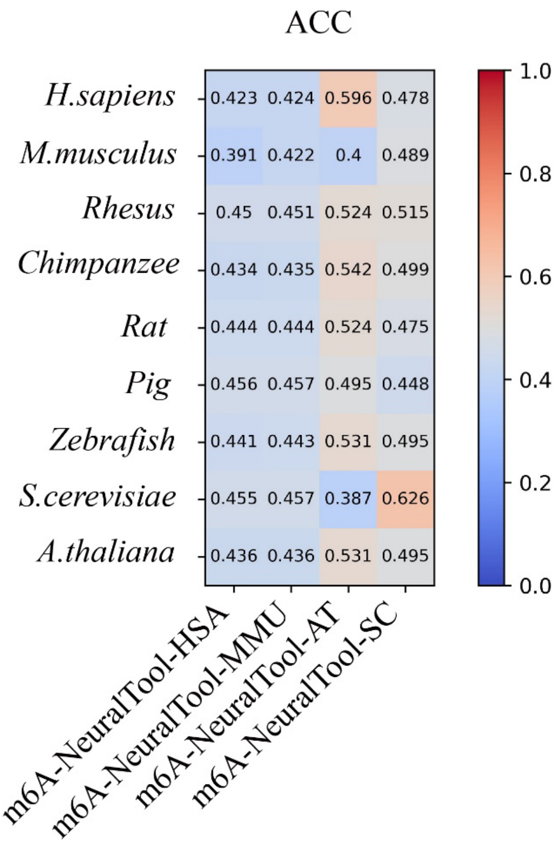

Figure S8. Cross-Species performance comparison of m6A-NeuralTool predictor on independent datasets of 9 species.
